# Supplementary material for: MetaTiME integrates single-cell gene expression to characterize the meta-components of the tumor immune microenvironment
Source: Nat Commun. 2023 May 6;14:2634. doi: 10.1038/s41467-023-38333-8 (PMC10164163; doi:10.1038/s41467-023-38333-8)
Supplement: Supplementary file 1 — Supplementary Information [file 41467_2023_38333_MOESM1_ESM.pdf]

Supplementary Information

MetaTiME Integrates Single-cell Gene Expression to Characterize the Meta-components of the Tumor Immune Microenvironment

Yi Zhang[1,2], Guanjue Xiang[1,2], Alva Yijia Jiang[1], Allen Lynch[1,2], Zexian Zeng[1,2], Chenfei Wang[1,2], Wubing Zhang[1,2], Jingyu Fan[1,2], Jiajinlong Kang[1], Shengqing Stan Gu[3], Changxin Wan[1,2], Boning Zhang[1,2], X. Shirley Liu[1,2,4]*, Myles Brown[3,4*], Clifford A Meyer[1,2,4]*

1. Department of Data Science, Dana-Farber Cancer Institute, Boston, MA 02215, USA
2. Department of Biostatistics, Harvard T.H. Chan School of Public Health, Boston, MA 02215 USA.
3. Department of Medical Oncology, Dana-Farber Cancer Institute, Boston, MA 02215, USA.
4. Center for Functional Cancer Epigenetics, Dana-Farber Cancer Institute, Boston, MA, USA

*Corresponding author. Email: cliff_meyer@ds.dfci.harvard.edu
*Co-corresponding author. Email: myles_brown@dfci.harvard.edu
*Co-corresponding author. Email: xsliu.res@gmail.com

Supplementary Figures

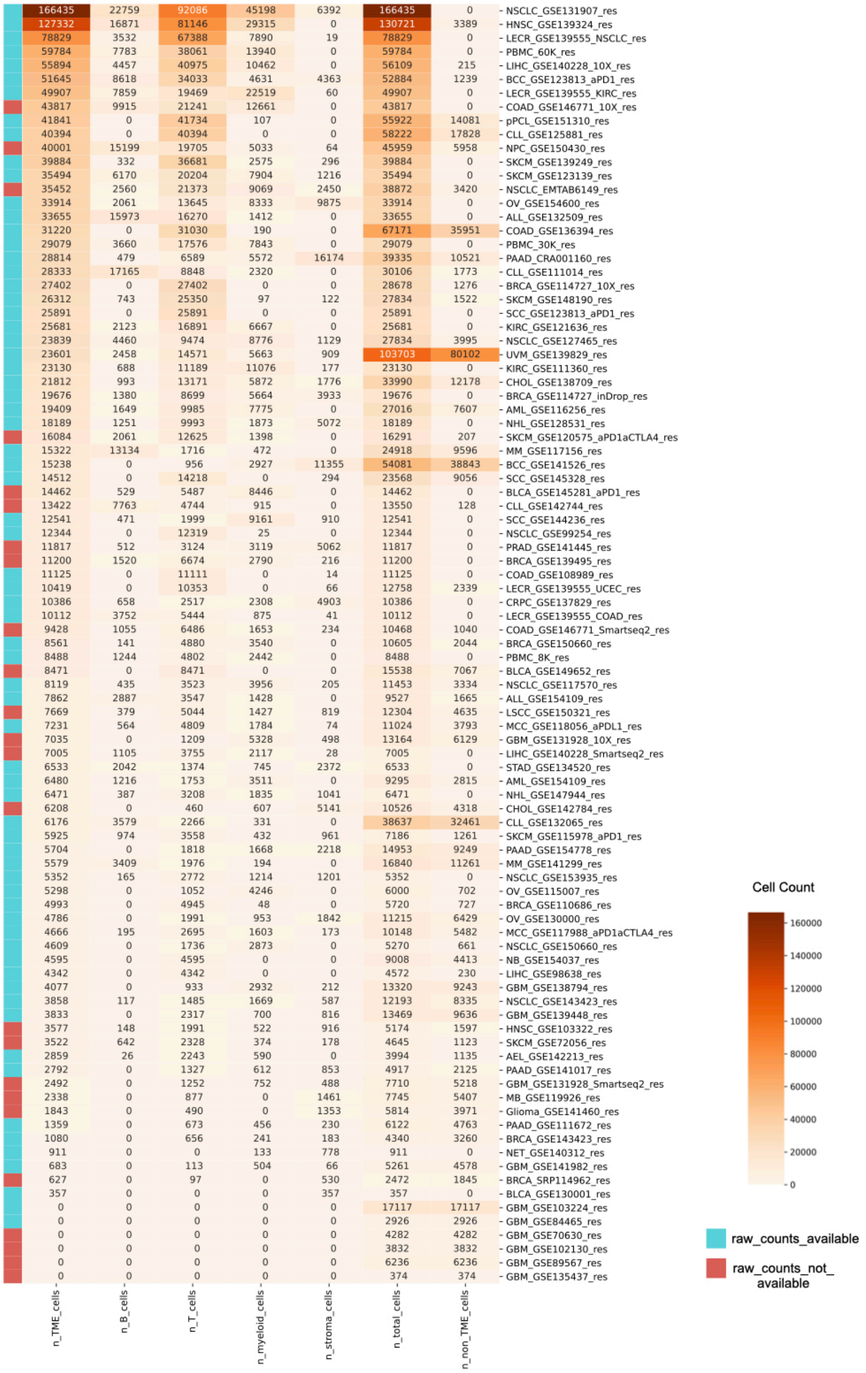

Supplementary Fig.1. Cell statistics of public tumor scRNA-seq collection. We utilized the curated cell type annotation from TISCH to summarize the number of cells in each category. MetaTiME includes immune cells and tumor microenvironment cells from each dataset, and excludes the malignant cells with low reproducibility across datasets. Datasets with raw counts available are marked in blue while datasets with only continuous TPM available are marked in red.

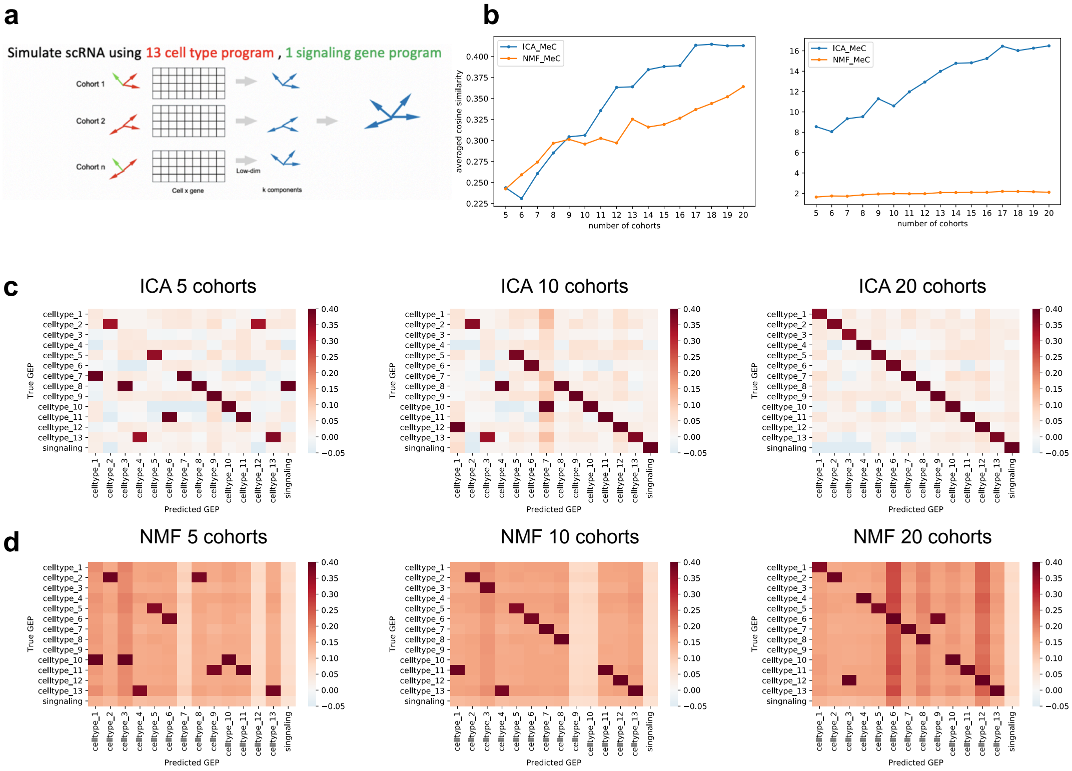

Supplementary Fig.2. Simulating scRNA data with transcriptional gene programs. Two dimension reduction methods were benchmarked using simulated scRNA data **(a)** Simulated gene expression program includes 13 cell type-specific gene programs and one pan-cell type gene program, mimicking common signaling pathways in single-cell RNA-seq data. The MetaTiME method was applied on the simulated cohorts to extract meta-components (MeCs) which were later compared with the pre-defined gene programs using correlation statistics. **(b)** Numbers of cohorts were tested to assess the robustness of the meta-components. The similarity between the recovered gene programs and the true gene programs (GEP) increases with larger cohort numbers. The average similarity score of ICA-derived MeCs is higher than NMF-derived MeCs. **(c)** The pairwise similarity between true GEP and predicted GEP using ICA for 5, 10, 20 cohorts. **(d)** The pairwise similarity between true GEP and predicted GEP using NMF for 5, 10, 20 cohorts.

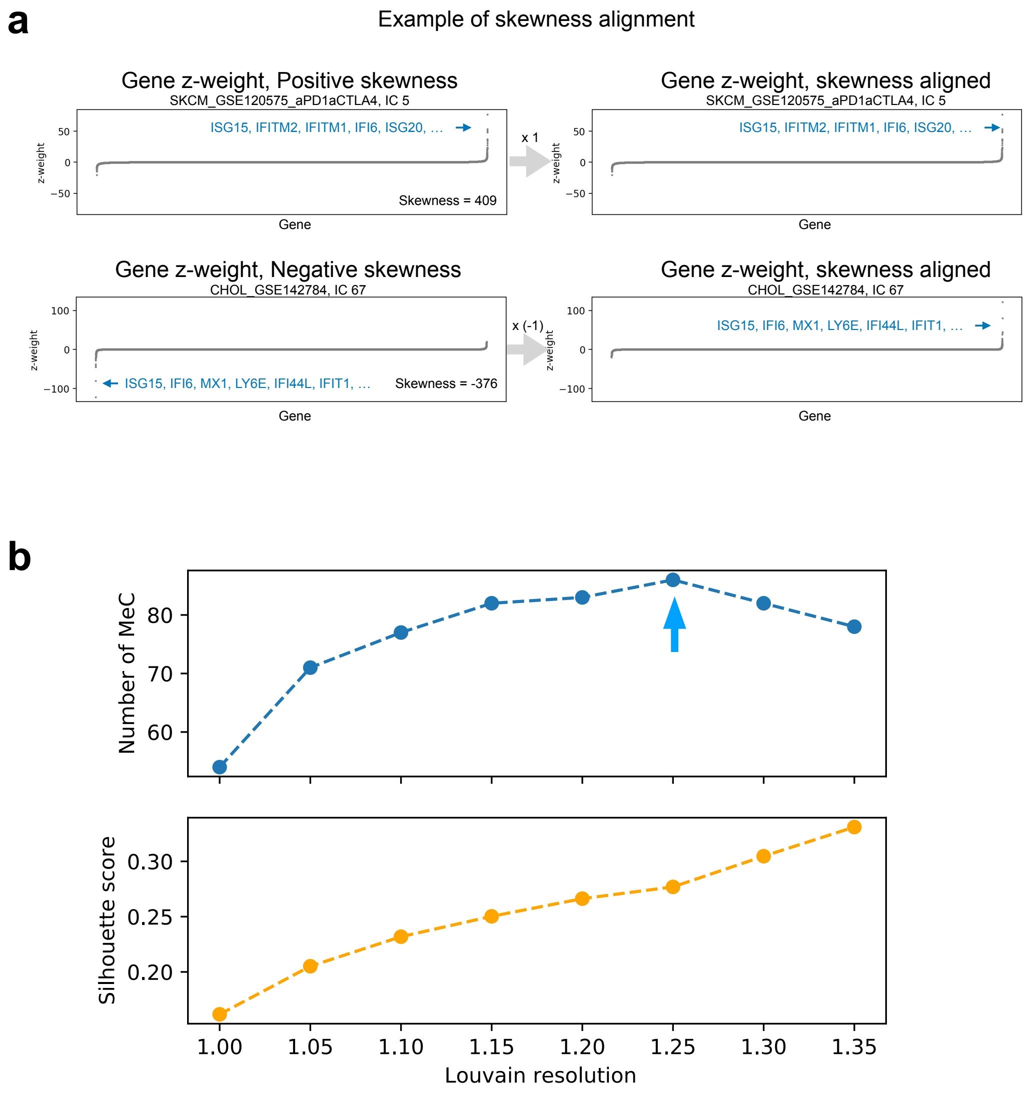

Supplementary Fig.3. Meta-component skewness and parameter tuning. (a) Genes most highly associated with a meta-component (MeC) tend to be biased to either significantly positive or significantly negative z-weights since the signs of the independent components are arbitrary. Examples of skewness of two MeCs, one with positive skewness (top row), the other with negative skewness (bottom row). MeCs with negative skewness are flipped by multiplying the gene weights by -1. **(b)** To determine the number of MeCs, we tested a range of resolution parameters in the community-detection based Louvain clustering algorithm. The Silhouette score increases as resolution increases. However, higher Louvain clustering resolution results in MeC clusters that are filtered as insufficiently reproducible across cohorts, which results in an overall decrease in the MeC number. The resolution parameter was thus selected to be 1.25, leading to 86 MeCs that optimize the tradeoff between cluster separation and reproducibility.

Supplementary Fig.4.

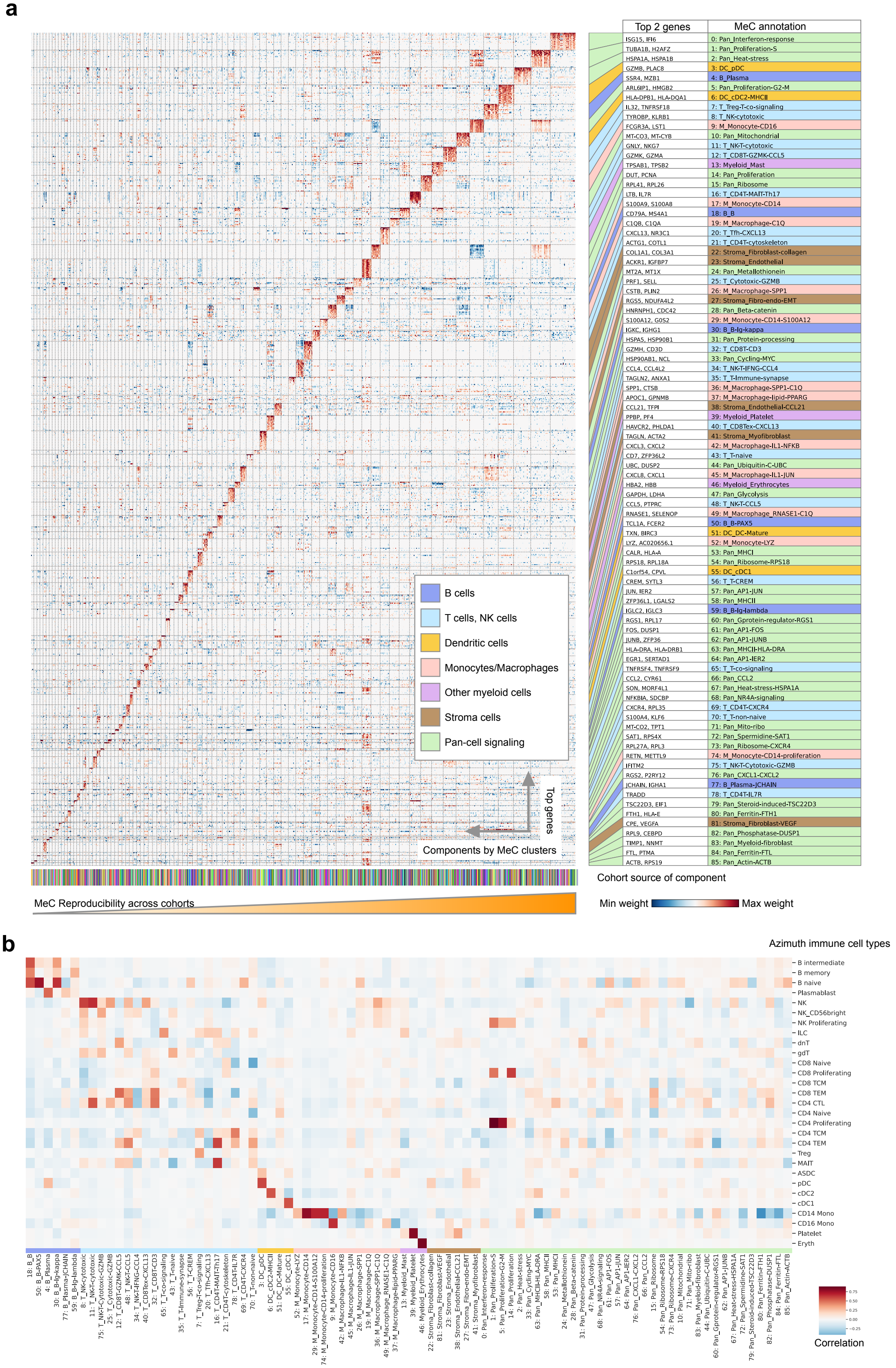

Supplementary Fig.4. Meta-components with annotation and comparison with known immune cell types. (a) All independent components clustered in MeCs are plotted using the normalized MeC gene z-weights, showing top featured genes in each MeC. Each row represents a gene, and each column represents an independent component. Components are ordered by MeC cluster assignment, and genes are ranked using MeC z-weights and deduplicated. The MeC annotation of each of the 86 MeCs are shown on the right with the top 2 genes marked. **(b)** Correlation between all functional MeCs and Azimuth by converting the marker list defined for immune cell types and subtypes to a list of weight 1.

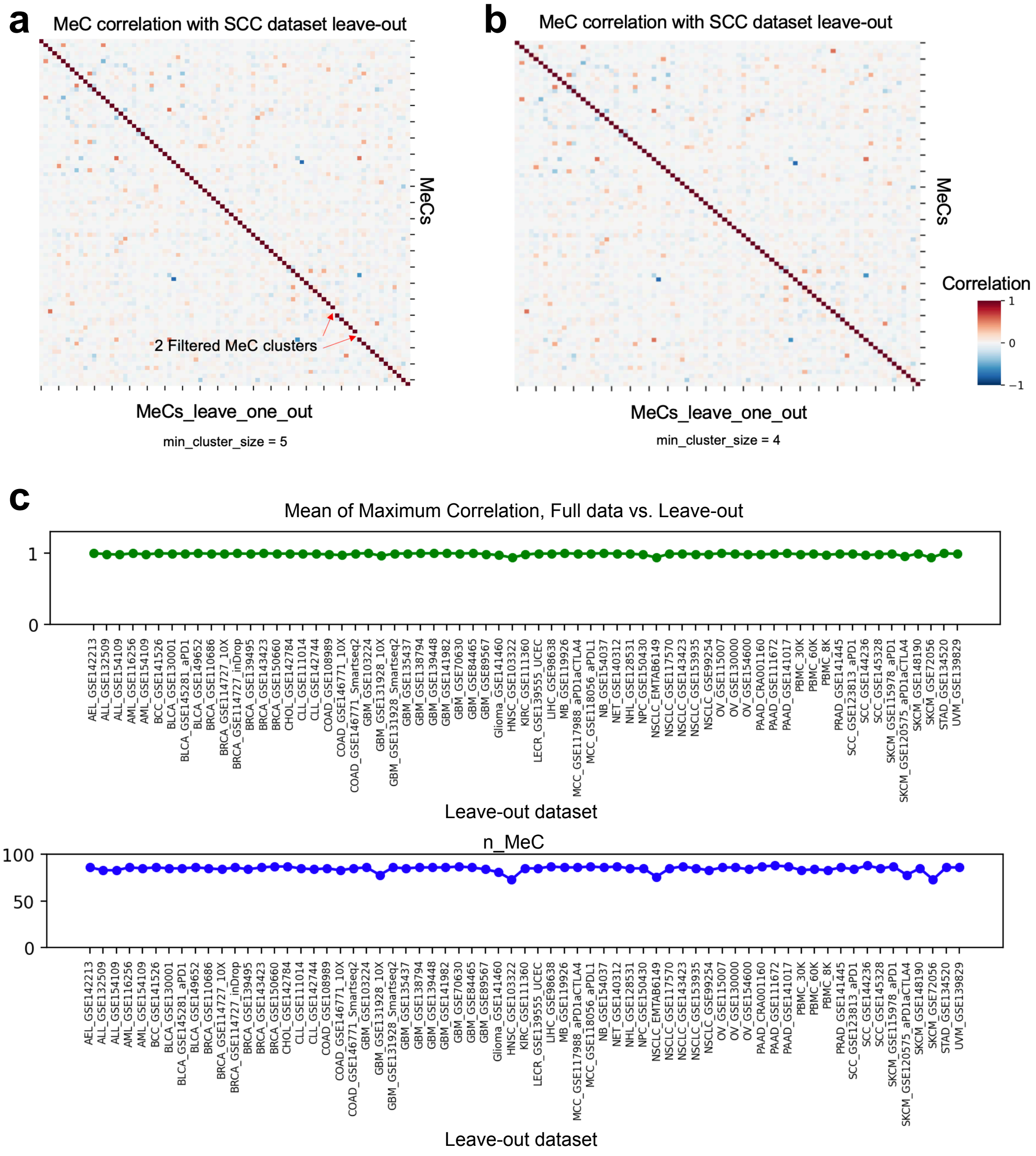

Supplementary Fig.5. Leave-one-out MeC training. (a) Correlation between MeCs called from the full set of datasets (row-wise) and the MeCs called leaving the SCC dataset out of MetaTiME training (column-wise), with the same cluster calling resolution parameter, and the minimum IC number in one cluster set to be 5 **(b)** Correlation between MeCs called from the full set (row-wise) and the MeCs called leaving the SCC dataset out (column-wise) when changing the minimum IC number in one cluster to be 4. **(c)** Leave-one-out MeCs compared to full-set MeCs, using the mean of row-wise and column-wise maximum correlation (top panel, mean max correlation=0.988), and number of MeCs (bottom, mean leave-one-dataset-out MeC number=84.64 compared to full-set MeC 86).

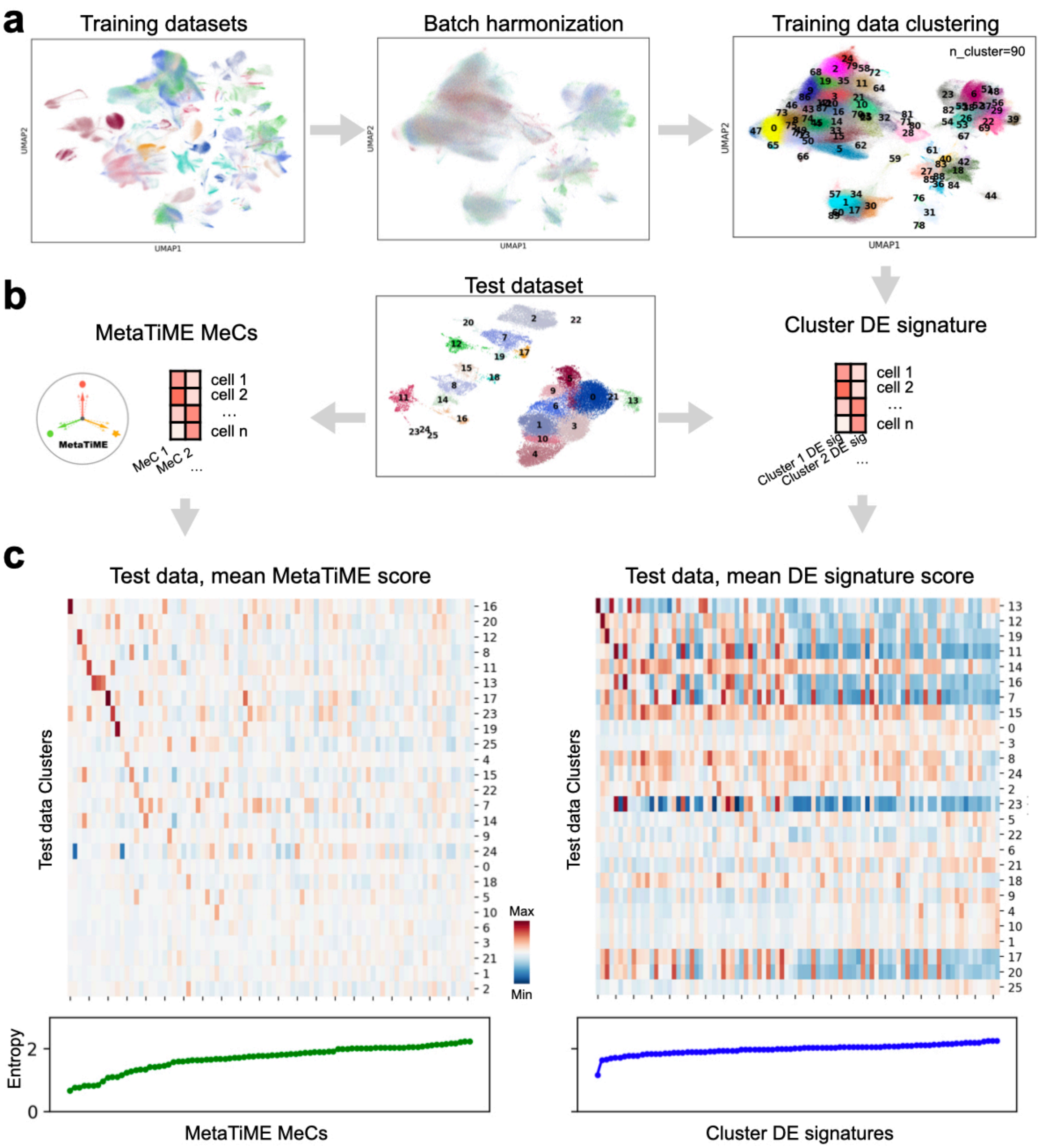

Supplementary Fig.6. Comparison of MetaTiME MeCs to signatures generated by clustering and differential gene expression analysis. (a) Direct integration of cells allows for inclusion of up to 21 datasets with the largest numbers of TME cells. Cells are harmonized across datasets as batches, clustered, and differential gene expression analysis performed to generate Cluster differential expression (DE) signatures. **(b)** Cluster DE signatures and MeC signatures are mapped to the test dataset where cells are also clustered. **(c)** The per-cluster mean scores of both signatures are plotted in heatmaps to assess whether the signatures are specific to test cell clusters or uniformly distributed, with entropy calculated to reflect the test cluster distribution of scores from MeC(left) and Cluster DE signatures (right).

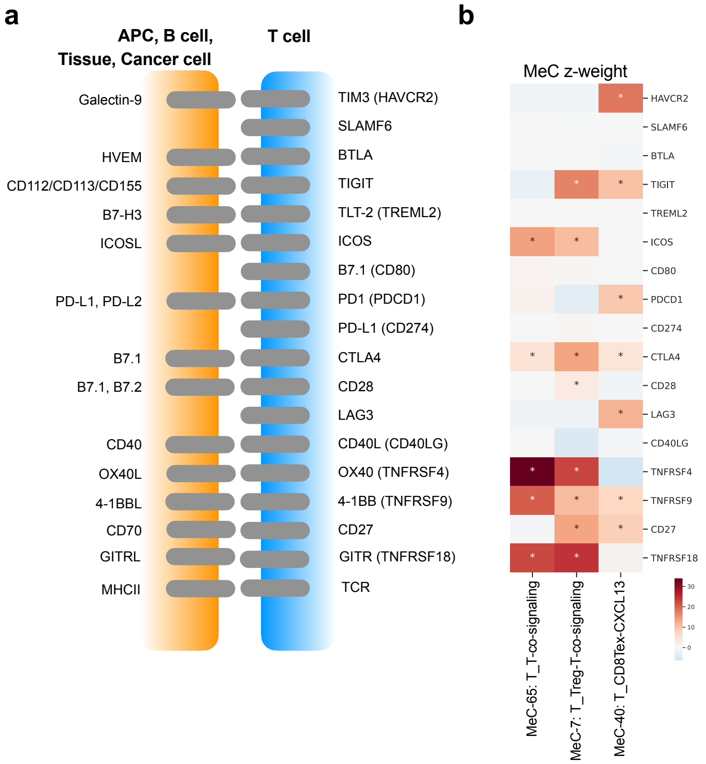

c

MeC-65: T_T-co-signaling	z-weight	rank
TNFRSF4	33.7	0
TNFRSF18	22.6	1
TNFRSF9	20.4	2
ICOS	14.0	9
CTLA4	4.5	108
TNFRSF8	4.3	117

MeC-7: T_Treg-T-co-signaling	z-weight	rank
HAVCR2	18.1	5
LAG3	11.8	17
TIGIT	9.8	28
PDCD1	9.1	33
CD27	8.0	42
TNFRSF9	7.2	48
CTLA4	4.2	100

MeC-40: T_CD8Tex-CXCL13	z-weight	rank
TNFRSF18	24.5	1
TNFRSF4	22.7	2
TIGIT	16.8	11
CD27	13.4	14
CTLA4	13.2	15
ICOS	10.6	21
TNFRSF9	10.3	24
CD28	3.2	127

Supplementary Fig.7. MetaTiME meta-components reveal co-signaling pathway genes in T cells. (a) Illustration of genes encoding membrane receptors on the T cell on the T cell co-stimulating and co-inhibitory pathways, with knowledge of interacting ligands from antigen presenting cells (APC), B cells, tissue cells or cancer cells. **(b)** Heatmap of meta-component z-weight of T co-signaling genes in three related T cell MeCs. Genes with significant contributions to the MeC are marked with a star. **(c)** z-weight and ranking of T co-signaling genes in three related T cell MeCs. Source data are provided as a Source Data file.

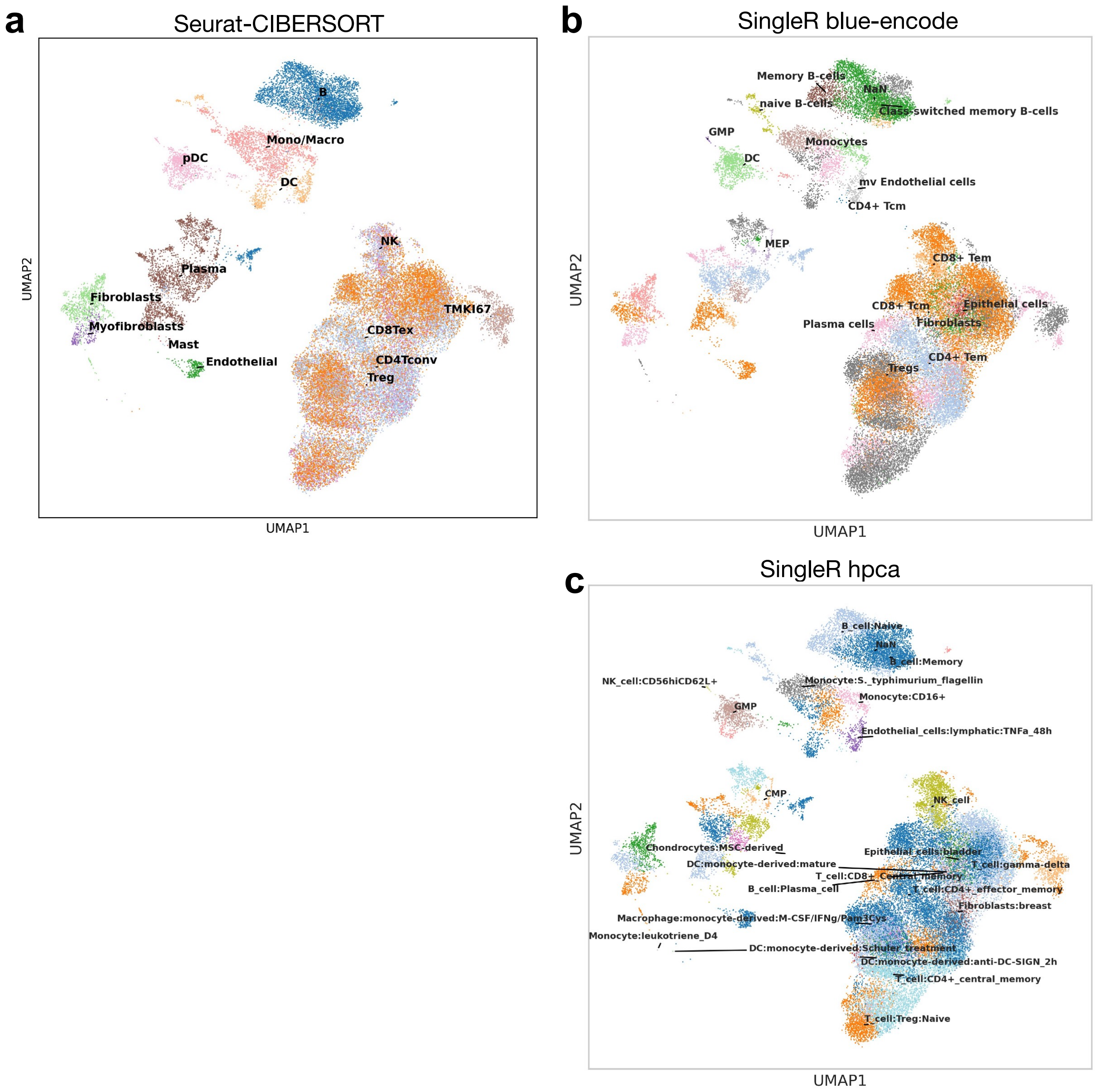

Supplementary Fig.8. Automatic annotation using existing methods and marker panels. (a). Seurat annotated results when using the CIBERSORT panel, as implemented in MAESTRO. **(b).** SingleR annotated results using the immune related panel Blueprint ENCODE (blue-encode). **(c).** SingleR annotated results using the immune related panel Human Primary Cell Atlas (hpca).

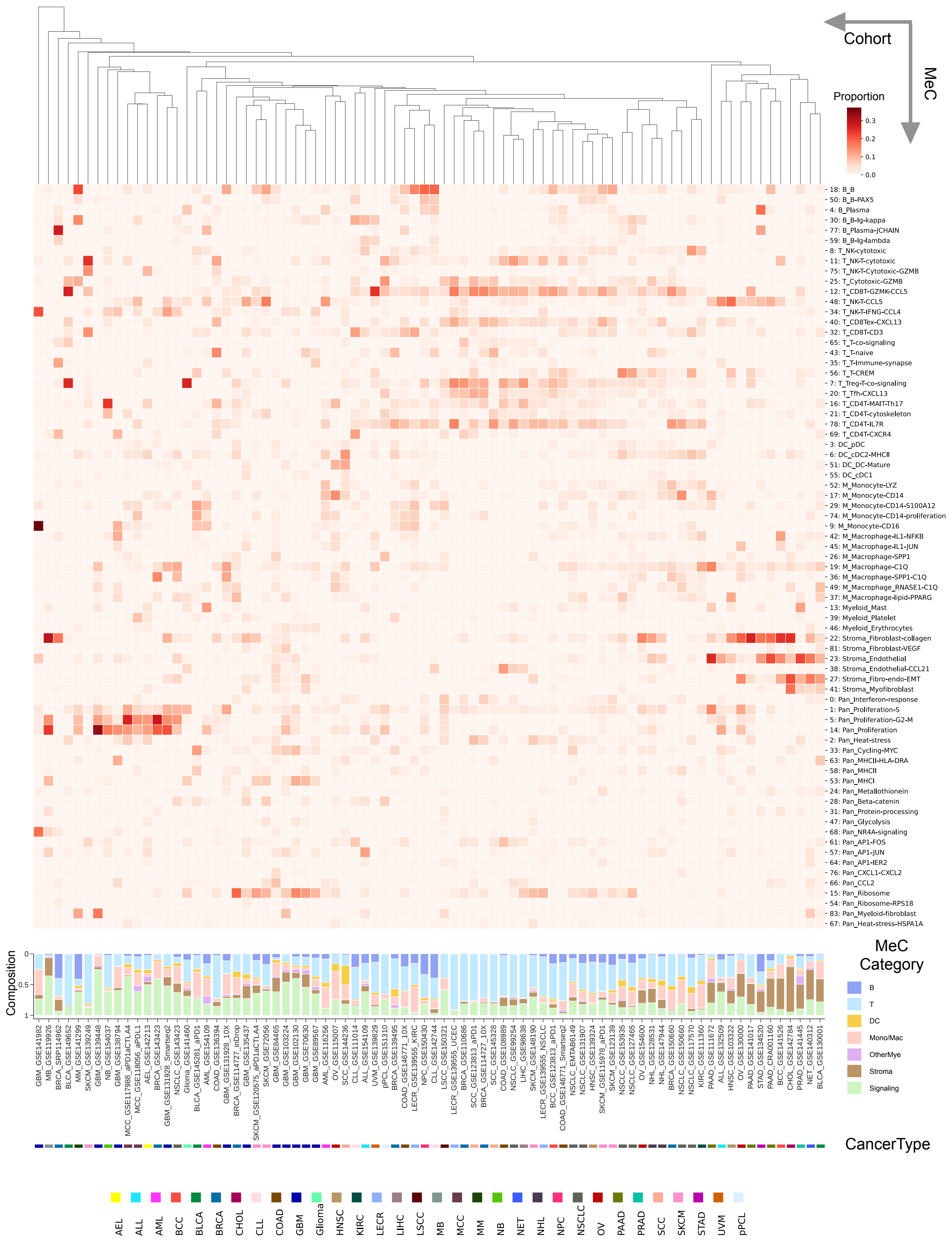

Supplementary Fig.9. Pan-cancer annotation of cell state composition in the full tumor scRNA datasets. Top: heatmap showing cell proportions in each dataset. Bottom: bar plot showing cell state composition of tumor microenvironment for tumor scRNA dataset cell states. The proportion of cell states from the same MeC category are aggregated. Source data are provided as a Source Data file.

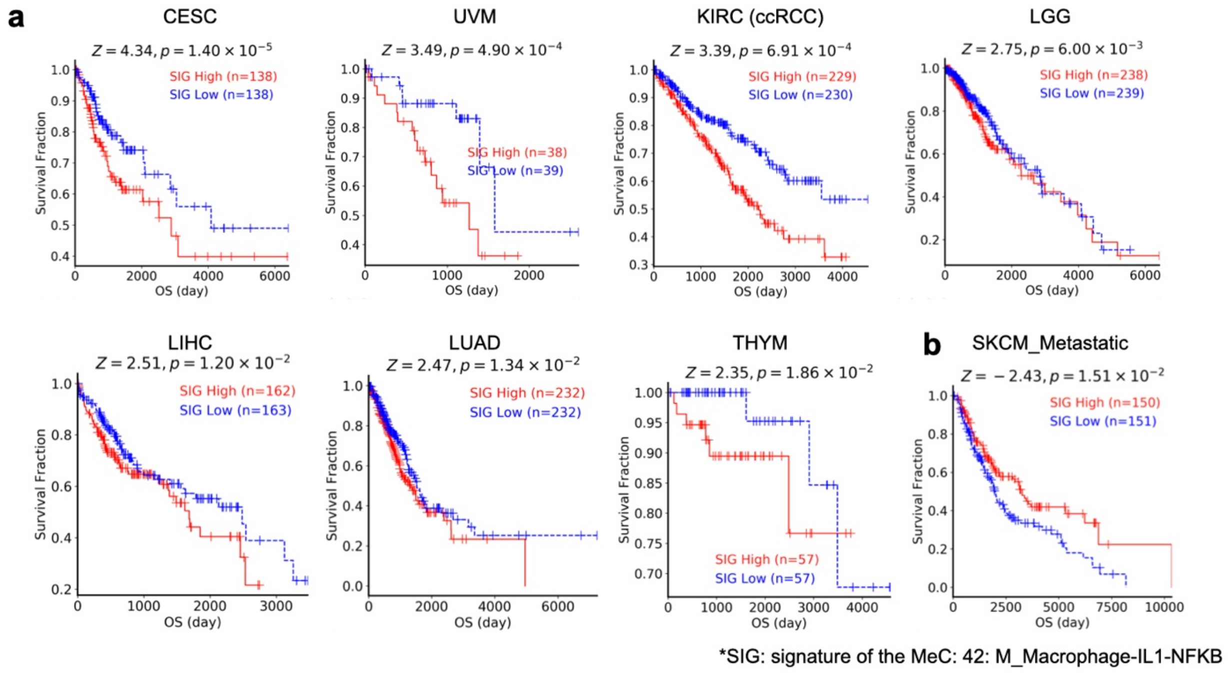

Supplementary Fig.10. TCGA survival prognosis of MeC representing IL1-activated macrophages. Each sample in TCGA is evaluated using top genes in MeC-42 “M_Macrophage-IL1-NFKB”, and patients are separated into two groups based on expression level of the signature. Survival fractions of patients are shown for all cancer types with significant separation. **(a)** In seven cancer types, patients with higher signatures have worse prognosis. Significance of the association was tested using Cox proportional hazards regression analysis. **(b)** In metastatic melanoma, patients with lower signatures have worse prognoses. The association was tested using Cox proportional hazards regression analysis. CESC: cervical squamous cell carcinoma and endocervical adenocarcinoma. UVM: uveal melanoma. ccRCC: clear cell renal cell carcinoma, named as KIRC in TCGA. LGG: low grade glioma. LIHC: liver hepatocellular carcinoma. LUAD: lung adenocarcinoma. THYM: thymoma. SKCM: skin cutaneous melanoma.

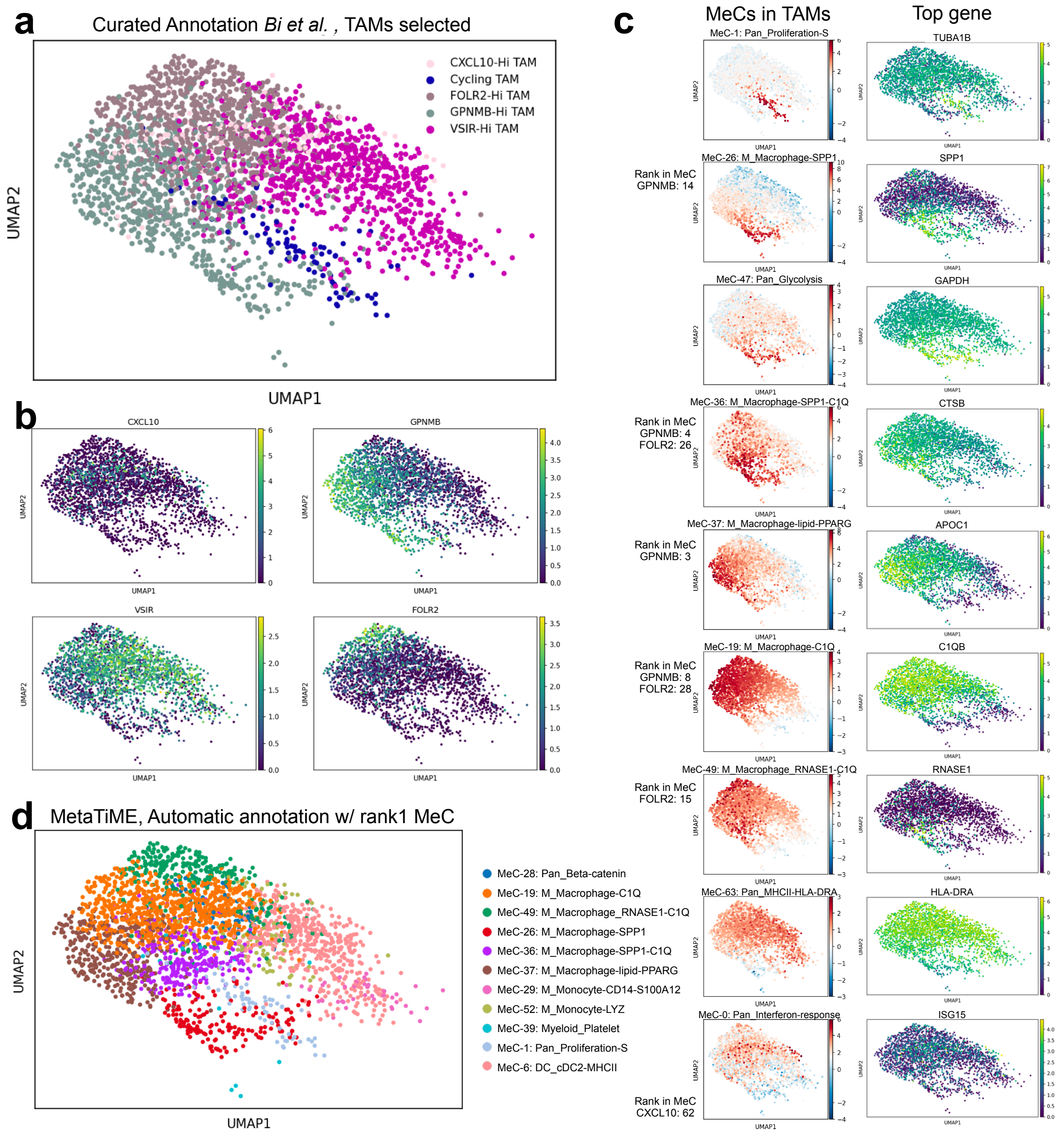

Supplementary Fig.11. Comparison with myeloid markers in a previous kidney cancer study. **(a)** Analysis was done on a kidney cohort with tumor associated macrophages. Cell types from the original study are shown. **(b)** Expression level of cell type markers used in the previous study. **(c)** Each row shows the scoring of a monocyte or macrophage related MetaTiME meta-component signature with expression of the marker gene top ranked by the meta-component. **(d)** A cluster-wise summary view of the most enriched MetaTiME meta-component. TAM: tumor associated macrophages.

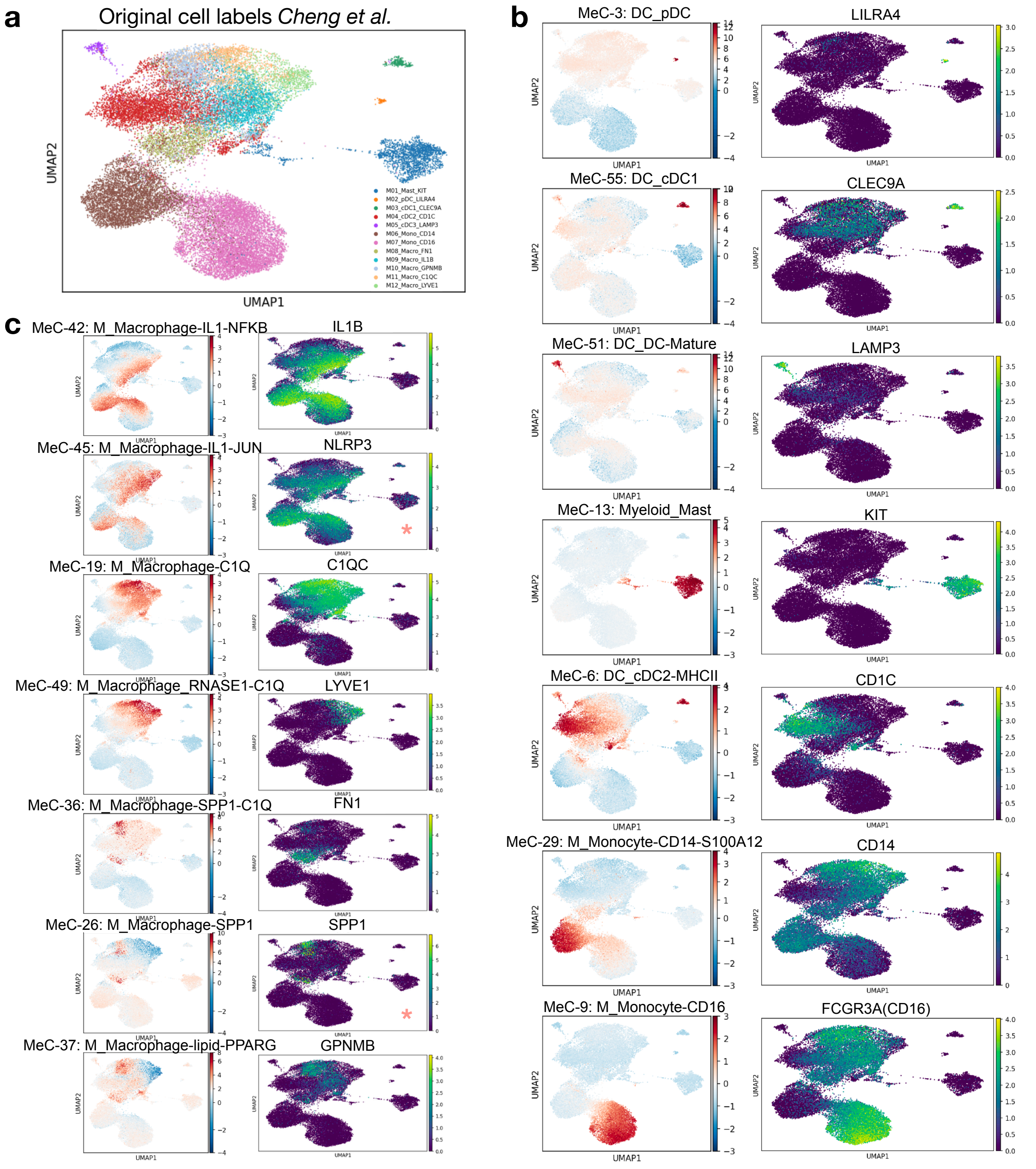

Supplementary Fig.12. Comparison with myeloid types defined by clustering and selected markers in a previous myeloid-focused kidney cancer study. (a) Analysis was done on a kidney myeloid cohort. Cell types from the original study are shown. **(b), (c)** Each row shows the scoring of a related MetaTiME myeloid meta-component signature (left) with expression of the marker gene from the original study (right). Genes with an orange star are marker genes from top of the MeC but not used in the original kidney cell types.

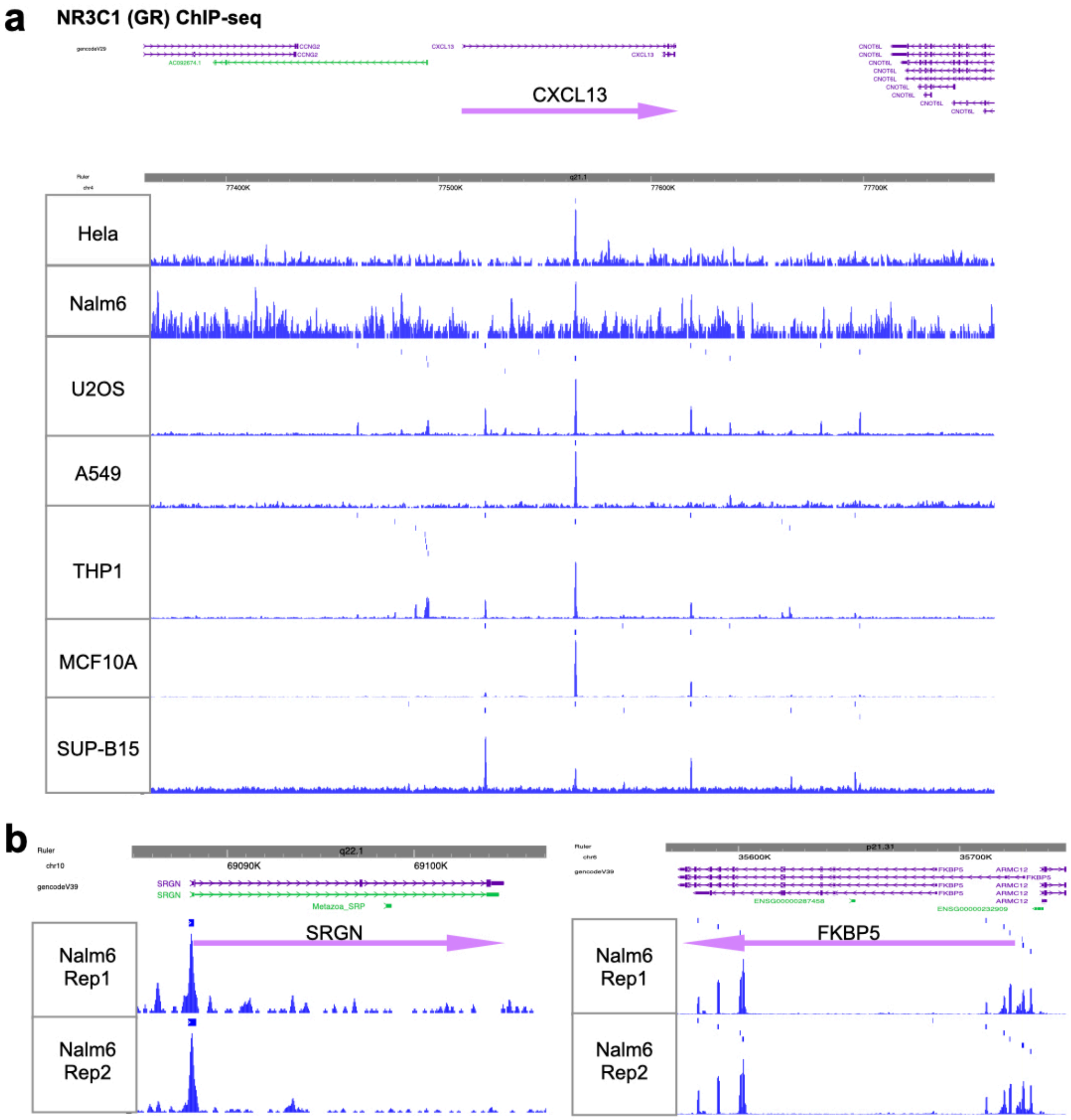

Supplementary Fig.13. ChIP-seq binding of NR3C1. (a) Glucocorticoid receptor (GR) ChIP-seq data from multiple cell lines including peaks and binding signals around CXCL13. **(b).**GR ChIP-seq data from Nalm6, a B cell leukemia line, binding at promoters of SRGN and FKBP5.
