# Supplementary material for: MetaTiME integrates single-cell gene expression to characterize the meta-components of the tumor immune microenvironment
Source: Nat Commun. 2023 May 6;14:2634. doi: 10.1038/s41467-023-38333-8 (PMC10164163; doi:10.1038/s41467-023-38333-8)
Supplement: Supplementary file 3 — Description of Additional Supplementary Files [file 41467_2023_38333_MOESM3_ESM.pdf]

Description of Additional Supplementary Files

Supplementary Data 1: Datasets and cell statistics.
Cells are counted based on value of the basic label in "assign_ident" from TISCH. Column "is_raw_counts_available" reflect whether raw data is raw counts with integer.

Supplementary Data 2: Meta-component interpretation.
Meta-component Annotation: Brief naming of each meta-component (MeC), with top 20, 100 genes by MeC z-weight. Annotation nonmeclature: "Category_CellState-Features". The first field before the underscore symbol marks 7 category of MeCs, 6 related to lineages: “B” for B cell, “T” for CD4T, CD8T, NK cells, “DC” for dendritic cell, “M” for monocytes and macrophages, “Myeloid” for other myeloid types besides monocytes and macrophages, “Stroma” for stromal cells, and the last category related to pancelltype signaling pathway: “Pan” for pan-cell signaling pathway.
Meta-component Enrichment: The significant enriched terms using various libraries using Enrichr and top 100 genes, as well as using GSEA and ranks of all genes.
Meta-component Regulator: Meta-component putative regulator based on either MeC z-weight or Lisa epigenetic siginificance. Factors in column TF_MeCLisa_top and TF_MeCLisa_top_1: TFs both highly weighted in MeC and significant in Lisa. Factors in colum TF_MeC_top: TFs significant only in MeC but not in Lisa binding. Factors in column TF_Lisa_top: TFs significant in Lisa binding but not MeC z-weight.
